# Effectiveness of alternative listening devices to conventional hearing aids for adults with hearing loss: a systematic review protocol

**DOI:** 10.1136/bmjopen-2016-011683

**Published:** 2016-10-27

**Authors:** David W Maidment, Alex B Barker, Jun Xia, Melanie A Ferguson

**Affiliations:** 1 National Institute for Health Research Nottingham Hearing Biomedical Research Unit, Nottingham, UK; 2 Division of Clinical Neuroscience, School of Medicine, University of Nottingham, Nottingham, UK; 3 Systematic Review Solutions Limited, Nottingham, UK; 4 Nottingham University Hospitals NHS Trust, Nottingham, UK

**Keywords:** OTOLARYNGOLOGY

## Abstract

**Introduction:**

Hearing loss is a major public health concern, affecting over 11 million people in the UK. While hearing aids are the most common clinical intervention for hearing loss, the majority of people that would benefit from using hearing aids do not take them up. Recent technological advances have led to a rapid increase of alternative listening devices to conventional hearing aids. These include hearing aids that can be customised using a smartphone, smartphone-based ‘hearing aid’ apps, personal sound amplification products and wireless hearing products. However, no systematic review has been published evaluating whether alternative listening devices are an effective management strategy for people with hearing loss.

**Methods and analysis:**

The objective of this systematic review is to assess whether alternative listening devices are an effective intervention for adults with hearing loss. Methods are reported according to the Preferred Reporting Items for Systematic reviews and Meta-analyses Protocols (PRISMA-P) 2015 checklist. Retrospective or prospective studies, randomised controlled trials, non-randomised controlled trials, and before-after comparison studies will be eligible for inclusion. We will include studies with adult participants (≥18 years) with a mild or moderate hearing loss. The intervention should be an alternative listening device to a conventional hearing aid (comparison). Studies will be restricted to outcomes associated with the consequences of hearing loss. We will search relevant databases to identify published, completed but unpublished and ongoing trials. The overall quality of included evidence will be evaluated using the GRADE system, and meta-analysis performed if appropriate.

**Ethics and dissemination:**

No ethical issues are foreseen. The findings will be reported at national and international conferences, primarily audiology, and ear, nose and throat, and in a peer-reviewed journal using the PRISMA guidelines.

**Review registration number:**

PROSPERO CRD4201502958.

**Strengths and limitations of this study**

- The protocol poses a clearly formulated research question identified and prioritised by key stakeholders in a recent James Lind
  Alliance Priority Setting Partnership for mild-to-moderate hearing loss.
- The systematic review will be the first to pool together evidence assessing the effectiveness of alternative listening devices to conventional hearing aids.
- Studies assessing analogue (as opposed to digital) hearing aids will be excluded, as they are an obsolete technology.
- We will only include studies with adults (≥18 years), since audiological characteristics differ substantially between children and adults.
- Grey literature and dissertation abstracts will not be searched.

## Introduction

Hearing loss affects over 11 million people in the UK (1 in 6 of the population), and is expected to rise to 15.5 million by 2030 given the ageing population profile.^1^ If untreated, hearing loss can make communicating with others difficult, which may lead to social isolation and withdrawal, mental illness (eg, anxiety, depression), and reduced quality of life.^2^ ^3^ Hearing loss is associated with an increased risk of dementia and mortality in older adults.^4^ ^5^ Unemployment rates are higher in people with hearing loss, costing the UK an estimated £24.8 billion in lost economic output each year.^6^ Consequently, hearing impairment is a major public health concern, having a significant impact on the individual, significant others and to society more generally.

The most prevalent degree of hearing loss can be defined as mild-to-moderate, affecting 92% of all adults with hearing loss in the UK.^1^ Hearing sensitivity can be assessed according to pure-tone thresholds across five different octave frequencies (0.25, 0.5, 1, 2, 4 kHz), with mild hearing loss indicated as a 20–40 dB hearing level (HL) and moderate as a 41–70 dB HL average in the better hearing ear.^7^ The most common clinical intervention for people with hearing loss is hearing aids, yet up to 60% of adults with hearing loss who would benefit from hearing aids do not take them up.^8^ ^9^ Furthermore, although the prevalence and severity of hearing loss gradually increases from the age of around 55 years, hearing aids are not typically adopted until people reach, on average, their mid-70s. The majority of first-time hearing aid users subsequently report that they have had a hearing problem for around 10 years or more before seeking help.^10^ Of particular relevance here is that the older people are when they receive hearing aids, the greater difficulties they may have adapting to and maintaining them.^10^ ^11^ Conversely, hearing aid fitting from an earlier age can result in substantial benefits (ie, more years of use, better use) compared with age-matched individuals fitted with hearing aids at a later age.^10^ It is therefore imperative that effective strategies are found that will promote the management of hearing loss from the earliest age possible, and so minimise the negative consequences associated with untreated hearing loss.

Conventional hearing aids do not restore hearing but make sounds more audible through electroacoustic amplification. Hearing aids are regulated medical devices that deliver sound into the ear canal via air or bone conduction. Bone conduction hearing aids and bone anchored hearing aids (BAHAs) are for people that have a conductive hearing loss (ie, sound cannot pass freely through the outer and middle ear) and so are unable to wear air conduction hearing aids. Rather than sound from the hearing aid being delivered through the ear canal, sounds are sent through the skull directly to the inner ear. Depending on the technology they use to process sounds, hearing aids can be described as ‘analogue’ or ‘digital’, and can be worn behind-the-ear (BTE), in-the-ear (ITE) or receiver-in-the-canal (RIC). In the UK, hearing aids can be obtained and fitted free of charge from a qualified hearing healthcare professional trained in audiology via the National Health Service (NHS) or a registered hearing aid dispenser in the private sector.

It may be taken for granted that hearing aids are an effective intervention for hearing loss. Indeed, a previous systematic review of literature published up to August 2004 suggested that hearing aids improve hearing-related quality of life, alleviating activity limitations and participation restrictions associated with hearing loss through improved communication. Nevertheless, the quality of the evidence was poor, with only two randomised controlled trials (RCTs) eligible to be included.^12^ This systematic review is currently being updated in a Cochrane review of RCTs and quasi-randomised controlled clinical trials.^13^

Recent advances in technology have also led to a rapid increase of innovative alternative devices that provide similar functionality (ie, amplification of sound) to conventional hearing aids.^14^ Alternative listening devices are of clear interest to patients and practitioners, reflected in the unanswered research question ‘Can new technologies replace hearing aids?’, ranked by patients and healthcare professionals as the fifth topmost ‘unanswered question’ in the recent James Lind Alliance Priority Setting Partnership for mild-to-moderate hearing loss.^15^ As a consequence, the Cochrane review^13^ alongside a registered systematic review on alternative listening devices will provide up-to-date high-level evidence on the effectiveness of a wide range of listening devices for adults with mild-to-moderate hearing loss.

Alternative devices currently available include: (1) ‘made-for-smartphone’ hearing aids, which allow users to adjust and personalise their hearing aid settings with their smartphone; (2) smartphone apps (Android and Apple OS X), whereby a smartphone or tablet computer can act as a hearing aid when paired with wireless Bluetooth earphones or earbuds; (3) self-fitting personal sound amplification products (PSAPs) that provide an ‘off-the-shelf’ alternative to hearing aids; (4) wireless hearing products that enhance the use of hearing aids so that they can be connected to additional electronic devices such as mobile phones, MP3 players or televisions via frequency modulation systems or Bluetooth. Alternative listening devices may be particularly appealing to users in comparison to conventional hearing aids because of their convenience and accessibility, providing additional functionality, such as listening to music, video gaming or watching television.^16^ Convenience and ease of access of interventions are key factors influencing rehabilitation decisions in people with hearing loss.^17–19^ Moreover, digital literacy skills continue to rise in 55–74 years old (2011=68%; 2013=76%; 2015=81%),^20^ suggesting that digital skills would not be a barrier to using the alternative devices described. Moreover, alternative listening devices may be an effective management strategy for hearing loss. For example, when compared with conventional hearing aids, smartphone-based hearing aid apps have been shown to provide similar levels of amplification, improved speech-in-noise performance and greater self-reported benefit.^21^ In addition, the use of alternative listening devices may promote earlier intervention, in addition to facilitating clinical help seeking.^22^ One potential explanation for these findings is that alternative listening technologies not only raise awareness of the consequences of hearing loss, but also increase self-efficacy (or confidence) to seek clinical rehabilitation.^22^

To date, no systematic review has been published that specifically evaluates the quality of evidence concerning whether alternative listening devices are an effective intervention for people with hearing loss. This is important because a high-quality, impartial body of evidence is necessary to determine whether alternatives could be adopted and/or recommended to people with hearing loss. There is also a strong clinical need to determine the effectiveness of alternative listening devices to conventional hearing aids, particularly given that, in the UK, the majority of adults with hearing loss have difficulties accessing them. One significant barrier to access is that up to 50% of adults who have hearing loss are not referred onwards to hearing healthcare professional by their general practitioner.^10^ Furthermore, at present, one clinical commissioning group in the UK no longer provides hearing aids for people with mild-to-moderate hearing loss.

The primary objective of this study is to systematically review existing evidence that assesses whether alternative listening devices to conventional hearing aids are an effective intervention for adults with hearing loss. We define alternative devices as both standalone products and devices that provide additional features to a conventional hearing aid. Secondary objectives are to assess feasibility (eg, accessibility, usability, acceptability, take-up, adherence) of alternative listening devices. It is anticipated that this review will provide an evidence base to help inform future feasibility and effectiveness trials on alternative devices, an approach consistent with the Medical Research Council's (MRC's) guidelines for evaluating complex healthcare interventions.^23^

## Methods and analysis

The methods of the systematic review are reported according to the Preferred Reporting Items for Systematic reviews and Meta-analyses Protocols (PRISMA-P) 2015 checklist.^24^ ^25^ Subheadings correspond to the recommended items to address in a systematic review protocol according to the checklist. The systematic review will be led by DWM, and specific roles of the named authors of the review protocol are specified in each section where appropriate.

### Eligibility criteria

The inclusion criteria are specified according to the Participant, Intervention, Comparison, Outcome and Setting (PICOS) study characteristics.

*Types of study*: Retrospective or prospective studies, RCTs, non-RCTS, and before-after comparison studies will be eligible for inclusion. Articles reporting expert opinions, practice guidelines, case reports, case series, conference abstracts and book chapters will be excluded.

*Participants*: We will include adults (≥18 years) with a mild or moderate hearing loss (average hearing threshold across octave frequencies 0.25–4 kHz ≥20 and ≤70 dB HL^7^), given that audiological characteristics differ substantially between children and adults.^26^ Studies that include both children (<18 years) and adults will not be included unless data are reported separately. If the hearing threshold is not specified, the study author will be contacted for further clarification. If hearing threshold data are not reported and cannot be obtained, studies will be included where the mean average hearing threshold reported falls within the range of either mild (between 20 and 40 dB HL) or moderate hearing loss (between 41 and 70 dB HL). Studies will be included if only qualitative descriptions of hearing threshold are provided with no audiometric data, but they will not be included in the meta-analysis. Bilateral and unilateral sensorineural, conductive and mixed hearing losses will be included.

*Intervention*: Any alternative listening device to a conventional hearing aid will be included. Alternative devices can be standalone products that are not regulated medical devices (eg, smartphone app, self-fitting PSAP) or devices that provide additional features to a conventional hearing aid (eg, ‘made-for-smartphone’ hearing aid, wireless hearing product). Alternative listening devices should aim to improve hearing and communication outcomes in people with hearing loss through the amplification of external sound sources.

*Comparison*: Comparisons can be inactive (eg, unaided, no treatment, usual care, waiting list) or active (eg, conventional hearing aid, BAHA, other alternative listening device). Conventional hearing aids are defined as a regulated medical device that delivers electroacoustic amplification via air or bone conduction, irrespective of where they are worn (BTE, ITE, RIC). Studies with analogue hearing aid instruments (as opposed to digital) will not be included. In 2000, the Modernising Hearing Aid Services programme was introduced, whereby all NHS patients are now provided digital hearing aids as standard. Analogue hearing aids are therefore an obsolete technology.

*Outcome measures*: As this review is concerned primarily with the effectiveness of alternative listening devices, studies will be restricted to outcomes associated with the consequences of hearing loss. Primary outcomes will include one or more of the following: (1) behavioural measures of speech intelligibility (eg, intelligibility of syllables, words or sentences presented in quiet or in noise); (2) hearing-specific health-related quality of life, where participation is the key domain, measured using any self-report questionnaire (eg, Hearing Handicap Inventory for the Elderly, HHIE^27^); and (3) adverse effects, reported by the patient as pain, discomfort, tenderness, skin irritation or ear infection as a consequence of device fitting. Secondary outcomes will include any of the following self-report outcomes: (1) health-related quality of life (eg, Health Utilities Index Mark 3, HUI-3^28^); (2) listening ability (eg, Abbreviated Profile of Hearing Aid Benefit, APHAB^29^); (3) cognition (eg, working memory); (4) adverse effect, noise-induced hearing loss (eg, due to overamplification from inappropriate hearing aid fitting); and (5) feasibility (eg, usability, adherence).

*Settings*: Any research settings will be included.

### Information sources

The search protocol and methods have been developed by a medical information specialist (Dr Farhad Shokraneh, Systematic Review Solutions Limited).

A systematic search strategy will be employed to identify articles that meet eligibility for inclusion. We will search CINAHL (via EBSCOhost), Cochrane Library, EMBASE (via Ovid SP), MEDLINE (via Ovid SP), PubMed, Scopus, Citations Indexes of Web of Science, ISRCTN Registry, ClinicalTrials.Gov and WHO International Clinical Trials Registry Platform (ICTRP) to identify published, completed but unpublished and ongoing trials. All database searches will be completed in 1 day and with no time, language, document type or publication status limitations.

In addition, hand searching the past 6 months of publications from key audiology journals will be undertaken to ensure that any recently published articles are identified. Additional information will be identified manually through snowballing of the reference lists from included studies, as well as screening of related articles by shortlisted authors, to identify any relevant articles that may not have been returned by the initial database searches. Searches will be repeated 1 month prior to submission for publication to ensure that any newly published studies are included. Contact with study authors will be permitted to ascertain whether any studies are ongoing. Manual searches and personal author contact will continue up to the end of the data collection phase.

At the end of study selection process, search strategies for each database will be reported in a reproducible and replicable way, and a PRISMA flow diagram will be presented.

### Search strategy

The search terms were collected based on free text and controlled vocabularies (Medical Subject Headings (MeSH), Excerpta Medica Tree (EMTREE) and CINHAL Headings), expert opinion, literature review and checking the test search results.

We will use the following search strategy for PubMed, which will be adapted for other databases:

(‘Hearing Loss’[MeSH] OR Hypoacus*[tiab] OR ‘Hearing Loss’[tiab] OR ‘Hearing Losses’[tiab] OR ‘Hearing Impairment’[tiab] OR ‘Hearing Impairments’[tiab] OR Deaf*[tiab] OR ‘Hearing Problem’[tiab] OR ‘Hearing Problems’[tiab]) AND (‘Hearing Aids’[MeSH:NoExp] OR ((Auditory[tiab] OR Hearing[tiab] OR Amplif*[tiab] OR Listening[tiab] OR Audible[tiab] OR Hearable[tiab] OR Aural[tiab] OR Audio*[tiab]) AND (Prosthes*[tiab] OR Device*[tiab] OR Aid[tiab] OR Aids[tiab] OR Product[tiab] OR Products[tiab])) OR ‘Ear Mold’[tiab] OR ‘Ear Molds’[tiab] OR Conventional[tiab] OR Unaided[tiab] OR ‘No Treatment’[tiab] OR ‘Control Group’[tiab] OR ‘Usual Care’[tiab] OR ‘Waiting List’[tiab] OR Waitlist[tiab] OR ‘Treatment as Usual’[tiab] OR ‘Usual Treatment’[tiab] OR Routine[tiab] OR ‘Care as Usual’[tiab]) AND (‘Wireless Technology’[MeSH] OR ‘Smartphone’[MeSH] OR ‘Mobile Applications’[MeSH] OR Alternative*[tiab] OR Premium[tiab] OR Digital[ti] OR Smartphone*[tiab] OR ‘Smart Phone’[tiab] OR ‘Smart Phones’[tiab] OR iPhone[tiab] OR Wireless[tiab] OR ‘FM System’[tiab] OR ‘Frequency Modulation’[tiab] OR Bluetooth[tiab] OR ‘Assistive Listening’[tiab] OR ‘Mobile App’[tiab] OR ‘Mobile Apps’[tiab] OR ‘Mobile Application’[tiab] OR ‘Mobile Applications’[tiab] OR ‘Self-Fitting’[tiab]) AND (‘Clinical Trials as Topic’[MeSH] OR Randomized Controlled Trial[pt] OR Controlled Clinical Trial[pt] OR Pragmatic Clinical Trial[pt] OR Clinical Trial[pt] OR Observational Study[pt] OR Multicenter Study[pt] OR ‘Non-Randomized Controlled Trials as Topic’[MeSH] OR Randomized[tiab] OR Randomised[tiab] OR Nonrandomized[tiab] OR Nonrandomised[tiab] OR ‘Quasi Experimental’[tiab] OR Placebo[tiab] OR Randomly[tiab] OR Trial[tiab] OR Trials[tiab] OR RCT*[tiab] OR Groups[tiab] OR ‘Cross-Over Studies’[MeSH] OR ‘Cross Over’[tiab] OR Crossover[tiab] OR ‘Controlled Before-After Studies’[MeSH] OR ‘Before After’[tiab] OR ‘Before and After’[tiab] OR ‘CBA Study’[tiab] OR ‘CBA Studies’[tiab] OR ‘Cohort Studies’[MeSH] OR Cohort[tiab] OR Concurrent[tiab] OR Incidence[tiab] OR ‘Follow Up’[tiab] OR Followup[tiab] OR Longitudinal[tiab] OR Prospective[tiab] OR Retrospective[tiab] OR ‘Case-Control Studies’[MeSH] OR ‘“Case Control”’[tiab] OR ‘Case Comparison’[tiab] OR ‘Case Compeer’[tiab] OR ‘Case Base’[tiab] OR Retrospective[tiab]).

### Data management (DWM and ABB)

DWM will be responsible for data management using Covidence online software (https://http://www.covidence.org/). All identified articles will be recorded electronically, and can be tracked throughout the data screening and extraction process. Reasons for exclusion will be noted and included articles allocated a unique study ID code so that the record can be linked to the corresponding full text and data collection sheet.

### Selection process (DWM, ABB and MAF)

DWM and ABB will independently screen all relevant references to decide eligibility according to the inclusion and exclusion criteria by reading the title and abstract. The full text will be obtained for articles that appear to meet the inclusion criteria or where there is any uncertainty (ie, insufficient information to make a clear decision). Where necessary, study authors will also be contacted for additional information to resolve questions concerning eligibility. Discrepancies will be adjudicated by MAF.

### Data collection process (DWM, ABB, JX and MAF)

We plan to use a standardised data collection form constructed via Covidence. Detailed guidance notes will be devised by DWM prior to starting the review. The guidance notes and data collection form will be piloted by DWM and ABB, and revised if necessary before the review to ensure consistency. Data collection will be conducted by DWM and ABB independently, but in duplicate for every record included. Study authors will be contacted to resolve any uncertainties. MAF and JX will sample a subset (∼10%) of data collection sheets for each author to confirm consistency of approach. All authors will resolve any disagreement through discussion.

### Data items

The data collection form will include a list of fields given in table 1, consisting of study type, type of intervention and comparison, outcome measures and statistical tests. If any information is not reported, this will be recorded in the corresponding field. We will contact the corresponding author of any included study to obtain missing data. If data can only be estimated, this will be approximated from figures.

**Table 1:** Data items for the systematic review of trials on the effectiveness of alternative listening devices to conventional hearing aids

General information	Study ID
	Study title
	Reference citation
	Corresponding author and contact details
	Date of publication
Study eligibility	Peer reviewed
	English as first language
	Type of study
	Randomised controlled trial
	Non-randomised controlled trial
	Before and after studies
	Other
	Participants
	Age range
	Gender
	Hearing loss
	Health-related comorbidity
	Type of intervention (all arms)
	Types of comparison
	Types of outcome
Primary outcome domains	Speech intelligibility
	Hearing-specific health-related quality of life
	Adverse effects (pain)
Secondary outcome domains	Health-related quality of life
	Listening ability
	Cognition
	Adverse effect (noise-induced hearing loss)
	Feasibility
Results and statistical tests	
Risk of bias assessment (see Risk of bias in individual studies section)	
Other information (this optional field will be used to record further comments that may be deemed informative)	

### Risk of bias in individual studies (DWM and ABB)

DWM and ABB will independently assess risk of bias of each included study with the Cochrane Risk of Bias Tool, which rates the studies as ‘high risk’, ‘low risk’ or ‘unclear risk’ in the following six domains: sequence generation, allocation concealment, blinding of participants and outcome assessment, incomplete outcome, selective reporting and other bias. The overall quality of the evidence will be evaluated using the GRADE system.^30^ Scores on five principal domains will be used to assess the quality of the evidence: (1) limitations in design; (2) inconsistency of results; (3) indirectness of the evidence; (4) imprecision of results and (5) a high probability of publication bias. The quality of the data starts at ‘high’ and reduces by a level for each of the factors not met.

### Data synthesis (DWM and JX)

For binary data, we will calculate risk ratio with 95% CI where possible. For continuous data, we will calculate mean difference (MD) with 95% CI where the studies use the same outcome measures; otherwise, we will instead use standardised MD with 95% CI. If summary effects are reported, we will analyse these using generic inverse variance and report the effect estimates with its 95% CI.

In the absence of meta-analysis, primary and secondary outcomes will be assessed at the individual study level, with the main study findings examined through narrative synthesis.

### Assessment of heterogeneity

Heterogeneity across studies will be examined using I^2^ statistic. If I^2^ is >50%, we will explore potential statistical, clinical or methodological causes for heterogeneity through subgroup analysis. We will not pool studies if I^2^ exceeds 60%,^31^ but will present the data through narrative synthesis. For robustness, both random-effects and fixed-effects models will be used on the primary outcome measures.

### Assessment of reporting biases

Funnel plots will be used to estimate the influence of unpublished papers on the overall effects (ie, publication bias).

### Subgroup analysis

If heterogeneity is identified, subgroup analyses will assess the impact of participant age, gender, degree of hearing loss and the presence of comorbidity. Age will be defined as older adults (≥55 years) and younger adults (<55 years) since people with age-related hearing loss are typically 55 years and older, which is also the age eligibility threshold adopted by the services specification developed by the UK Department of Health.^32^ If a study reports males and females separately, it will contribute to both subgroups. If a study includes only one gender, it will be entered entirely into the appropriate subgroup. If a study reports a mixed group, it will not be included in the subgroup analysis. Degree of hearing loss will be classified according to better ear hearing thresholds as either mild or moderate. Comorbidity will be defined as any additional disease or disorder (behavioural, mental) co-occurring with hearing loss. Each comorbid disorder must be reported separately to contribute to the appropriate subgroup for analysis.

### Ethics and dissemination (DWM and MAF)

No ethical issues are foreseen. The finding will be reported at national and international conferences and meetings, primarily audiology and ENT (DWM), and in a peer-reviewed journal using the PRISMA guidelines (http://www.prisma-statement.org/; DWM and MAF). A public and patient involvement representative will be involved in disseminating the outcomes of the review through print and events aimed at non-specialist audiences.
